# The balance of crystalline and amorphous regions in the fibroin structure underpins the tensile strength of bagworm silk

**DOI:** 10.1186/s40851-021-00179-7

**Published:** 2021-07-26

**Authors:** Nobuaki Kono, Hiroyuki Nakamura, Ayaka Tateishi, Keiji Numata, Kazuharu Arakawa

**Affiliations:** 1 Institute for Advanced Biosciences, Keio University, 403-1 Nihonkoku, Daihouji, Tsuruoka, Yamagata Japan; 2 Spiber Inc., 245-1 Mizukami, Kakuganji, Tsuruoka, Yamagata Japan; 3 RIKEN Center for Sustainable Resource Science, 2-1 Hirosawa, Wako, Saitama, Japan; 4 Department of Material Chemistry, Kyoto University, Kyotodaigaku-Katsura, Nishikyo-ku, Kyoto, Japan

**Keywords:** Bagworm, Silk, H-fibroin, Transcriptome, Mechanical property, Fibroin architecture

## Abstract

Protein-based materials are considered versatile biomaterials, and their biodegradability is an advantage for sustainable development. Bagworm produces strong silk for use in unique situations throughout its life stages. Rigorous molecular analyses of *Eumeta variegata* suggested that the particular mechanical properties of its silk are due to the coexistence of poly-A and GA motifs. However, little molecular information on closely related species is available, and it is not understood how these properties were acquired evolutionarily or whether the motif combination is a conserved trait in other bagworms. Here, we performed a transcriptome analysis of two other bagworm species (*Canephora pungelerii* and *Bambalina* sp.) belonging to the family Psychidae to elucidate the relationship between the fibroin gene and silk properties. The obtained transcriptome assemblies and tensile tests indicated that the motif combination and silk properties were conserved among the bagworms. Furthermore, our analysis showed that *C. pungelerii* produces extraordinarily strong silk (breaking strength of 1.4 GPa) and indicated that the cause may be the *C. pungelerii* -specific balance of crystalline/amorphous regions in the H-fibroin repetitive domain. This particular H-fibroin architecture may have been evolutionarily acquired to produce strong thread to maintain bag stability during the relatively long development period of *Canephora* species relative to other bagworms.

## Background

Various fascinating protein-based materials found in nature are versatile representative biomaterials, and their biodegradability is an advantage [1]. Research on these high-performance materials can be of great help in artificial design and use efforts [2]. In particular, silk is a versatile biomaterial composed of silk fibroin that represents a unique combination of strength, toughness, and extensibility and is widely found in arthropods, mainly including members of the classes Insecta and Arachnida [3, 4]. The applications of silk have been optimized for clade-specific situations ranging from cocoon or egg sac formation to foraging, locomotion, or shelter production [3, 5]. The diversity of silk associated with the evolutionary and ecological adaptations of species may allow required physical properties to be designed and suggests a potential wide range of artificial applications.

Bagworms (Lepidoptera: Psychidae) are particularly strong silk-producing insects within Lepidoptera. Lepidopteran species produce cocoons using only thick, tough silk, and the composition of such silks has been well studied in a domesticated silkworm (Lepidoptera: Bombycidae) and a saturniid (Lepidoptera: Saturniidae) [6]. On the other hand, bagworm larvae not only produce a self-enclosing bag coated with silk and plant material but also use silk thread to hang their bag from the branch of a host tree [7, 8]. Although the cocoon hanging is known to be rare elsewhere, such as *Callosamia promethea* (family Saturniidae), it is one of the major features common to all bagworms. A detailed physical property analysis showed that the tensile strength of bagworm (*E. variegata*) silk (689 MPa) is nearly twice as high as that of domesticated silkworm silks (approximately 350 MPa) and shows up to half the average tensile strength of the dragline silk of orb-weaver spiders (approximately 1 GPa) [9, 10]. The available molecular information on *E. variegata* silk components, such as L/H-fibroin, sericin, fibrohexamerin, seroins, and protease inhibitors, has been increasing in recent years [11, 12]. In particular, we previously presented an *E. variegata* draft genome obtained via hybrid sequencing [13] and revealed all of the fibroin genes present based on a multiple-omics analysis [10]. The bagworm draft genome revealed a unique combination of repetitive motifs, polyalanine (A)_n_ sequences and alternating glycine-alanine (GA)_n_ sequences [10]. Most lepidopteran insects exhibit one of two such repetitive motifs in the fibroin gene. For example, saturniid fibroin genes contain an (A)_n_ motif, and the domesticated silkworm genes contain a (GA)_n_ motif [14]. Coexistence of the two motifs has not been found in other Lepidoptera, suggesting that this feature may underlie bagworm silk properties. Many examples of how mechanical properties change depending on the type of repetitive motifs are available [15, 16]. A more detailed understanding of the fibroin design rules based on comparative analysis among closely related species is required to produce artificial protein-based materials. However, the available molecular information on the Psychidae family is limited almost exclusively to *E. variegata*. Here, we performed transcriptome analysis and silk tensile test of *Canephora pungelerii* and *Bambalina* sp., which belong to the same subfamily (Oiketicinae, family Psychidae) as *E. variegata* to characterize the relationship between fibroin sequences and mechanical properties.

## Methods

### Bagworm and silk sampling

**Fig. 1:**
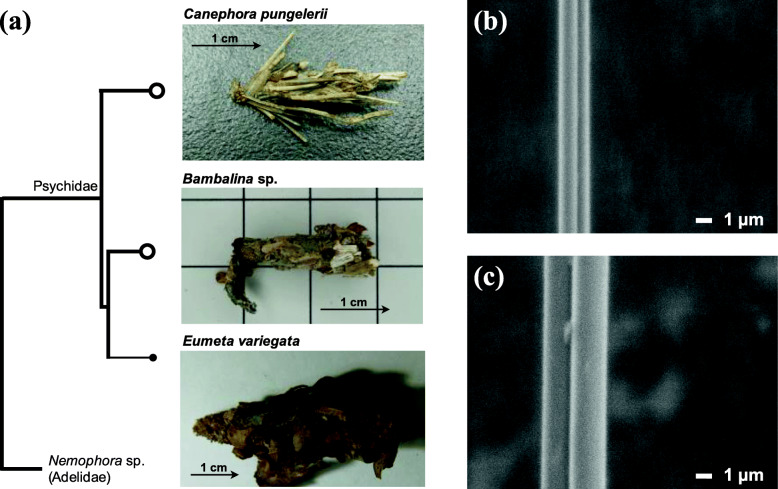
**a** Images of the bags of three bagworms (*C. pungelerii*, *Bambalina* sp., and *E. variegata*). The arrows indicate top to bottom when hanging. The phylogenetic tree was constructed based on orthologous genes from four lepidopterans, including *Nemophora* sp. (family Adelidae) as the root. **b** Scanning electron micrograph (SEM) image of *C. pungelerii* silk and (**c**) *Bambalina* sp. silk. The *C. pungelerii* silk is about twice as thin and the surfaces are both smooth

*C. pungelerii* and *Bambalina* sp. were collected from Yamagata Prefecture, Japan (May – Jul 2019) at the larval stage enclosed in a bag (Fig. 1a). The average size of the male *C. pungelerii* bag is 21.6 mm and the plant materials used for the bag are leaves and stems from various grasses. *Bambalina* sp. has a bag size of 37.3 mm and is known to use dead branches and leaves for its bag. These bagworms were identified based on the morphological characteristics of their bags and the sequence of cytochrome c oxidase subunit 1 (*COI*) based on the bagworm moth COI dataset [17]. These specimens were subjected to cDNA sequencing, and their silks were also used for the measurement of the mechanical properties (Fig. 1b, c). The silk samples were obtained from inside the bags. Since the samples were taken from the field, the nutritional conditions were not strictly controlled.

### Total RNA extraction

Total RNA extraction from bagworms was performed according to a field transcriptome protocol, as described previously [10, 18]. The bagworm larva was removed from its bag, immersed in 1 mL TRIzol Reagent (Invitrogen) and homogenized in a metal cone by using a Multi-Beads Shocker (Yasui Kikai). The extracted RNA was purified using an RNeasy Plus Mini Kit (Qiagen) with QIACube (Qiagen) automation. The quality of the purified total RNA was measured with a NanoDrop 2000 spectrophotometer (Thermo Scientific), and RNA integrity was estimated by electrophoresis using TapeStation 2200 with RNA Screen Tape (Agilent Technologies). Quantification was conducted using a Qubit Broad Range (BR) RNA assay (Life Technologies).

### Library preparation for cDNA sequencing

The cDNA library for Illumina sequencing was prepared using the NEBNext Ultra RNA Library Prep Kit for Illumina (New England BioLabs) following the manufacturer’s protocol. Total RNA samples (100 μg) were subjected to mRNA isolation by using NEBNext Oligo d(T)_25_ beads (skipping the second bead washing step). Double-stranded cDNA was synthesized using ProtoScript II Reverse Transcriptase and NEBNext Second Strand Synthesis Enzyme Mix. cDNA was end repaired using NEBNext End Prep Enzyme Mix before the ligation of the NEBNext Adaptor for Illumina and treatment with the USER enzyme. cDNA was amplified by PCR under the following conditions [20 μL cDNA, 2.5 μL Index Primer, 2.5 μL Universal PCR Primer, 25 μL NEBNext Q5 Hot Start HiFi PCR Master Mix 2x; 98 °C for 30 s and 12 cycles each of 98 °C for 10 s, 65 °C for 75 s and 65 °C for 5 min].

### Sequencing and de novo assembly

A cDNA sequencing was performed on a NextSeq 500 instrument (Illumina, Inc.) using 150-bp paired-end reads with a NextSeq 500 High Output Kit (300 cycles). The quality of the sequenced reads was assessed with FastQC. The de novo assembly of the bagworm sequences was performed with Bridger (v. 2014-12-01) (pair_gap_length = 0 and k-mer = 31).

### Gene annotation and fibroin curation

The assemblies were annotated with eggNOG-Mapper [19, 20]. Gene expression was estimated by using Kallisto version 0.42.2.1 as transcripts per million (TPM) values [21]. Homologous genes between the two bagworm species were identified according to bidirectional best hits (BBH) using a BLAST search with a 1.0e-20 threshold. Bagworm fibroin curation was conducted based on a previously reported Spidroin Motif Collection (SMoC) algorithm [13]. This strategy is effective for the curation of long gene sequences containing heavily repetitive regions, such as spider fibroin [22, 23], and has also been verified in *E. variegata* [10]. The Illumina short reads were assembled in a de Bruijin graph, and a BLAST search was carried out on the contigs from the N/C-terminal regions using *E. variegata* L/H-fibroin as the query (GBP72856.1 and GBP83861.1). The obtained terminal sequence candidates were used as seeds for screening short reads with an exact match of extremely large k-mers up to the 5′-end. The short reads were aligned on the 3′-side of the matching k-mer to build a position weight matrix (PWM). Based on a strict threshold, the seed sequence was extended until the next repeat unit appeared.

### Phylogenetics

Using HMMER (v 3.1b2) [24], the orthologous gene set was obtained from the bagworm assemblies and from *Nemophora* sp. (family Adelidae) as the root sample [25]. The 456 orthologous genes collected were aligned with MAFFT (v. 7.273) (mafft -auto- localpair-maxiterate 1000) [26] and then trimmed with trimAI (v. 1.2rev59) [27]. A bootstrap analysis was conducted with RAxML (v. 8.2.11), and the phylogenetic tree was visualized with FigTree version 1.4.3 (http://tree.bio.ed.ac.uk/software/figtree/).

### Measurement of mechanical properties

Surface morphology of the fibres were assessed by SEM (JCM 6000, JEOL Ltd., Tokyo Japan). Samples were mounted on an aluminum stub with a conductive tape backing and sputter-coated with gold for 1 min using a Smart Coater (JEOL) prior to SEM visualization at 5 kV. At least 8 individual mechanical stretching tests were performed for each silk type, with fibres were taken from two different bagworms for *C. pungelerii*, one bagworm for *Bambalina* sp.. The experimental setup was similar to those reported previously [10, 14]. Each fibre was attached to a rectangular piece of cardboard with a 5 mm aperture using 95% cyanoacrylate. The tensile properties of the fibres were measured using an EZ-LX universal tester (Shimadzu, Kyoto, Japan) with a 1 N load cell, at a strain rate of 10 mm/min (0.033 s-1) at 25 °C and 48% relative humidity. For each tensile test, the cross-sectional area of an adjacent section of the fibre was calculated based on the SEM images.

### Protein secondary structure data

We used values calculated in previous studies from the fibroin protein secondary structure data of spiders or closely related species. Spider silk structures were calculated by Raman spectromicroscopy [28], and the crystallinity of bagworm silk was estimated from the wide-angle X-ray diffraction (WAXD) profile [12].

## Results

### De novo transcriptome analysis of two bagworms and the corresponding phylogenetic tree

**Fig. 2:**
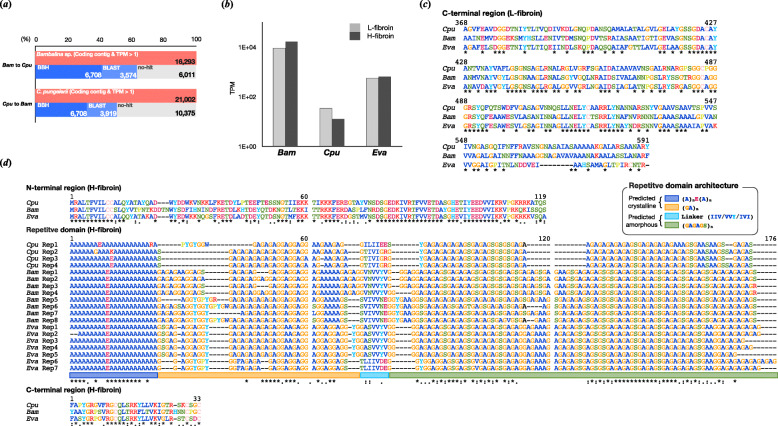
**a** The number of orthologous genes between *C. pungelerii* (*Cpu*) and *Bambalina* sp. (*Bam*) Among the assigned protein-coding contigs, 16,293 (*Bambalina* sp.) and 21,002 (*C. pungelerii*) genes had a TPM of 1 or higher, and the number of bidirectional best hits (BBH) between the two species was 6708. **b** Expression level of L/H-fibroin genes in the whole body of each individual. **c** Sequence alignment of the L-fibroin C-terminal region with *E. variegata* (*Eva*: GBP72856.1). **d** Sequence alignment of H-fibroin N/C-terminal regions and repetitive domains with *E. variegata* (GBP83861.1). The H-fibroin sequence was highly conserved among bagworms. The repetitive domain, represented as Rep, is divided into four motif regions: a poly-A region with glutamic acid in the centre (blue), a GA region (yellow), a linker region (light blue), and a GA region containing serine (green) as described in the box (repetitive domain architecture). From the previous SAXS analysis [12], it is predicted that the poly-A to GA region is the crystalline (β-sheet) region and the linker to GA region is the amorphous region

cDNA for transcriptome sequencing was synthesized from mRNA samples extracted from whole bagworm larvae. Illumina sequencing produced over 38 million 150-bp paired-end reads from each bagworm sample. These sequence reads were assembled, and 99,286 contigs (N50: 1815 bp) from *C. pungelerii* and 68,362 contigs (N50: 2017 bp) from *Bambalina* sp. were obtained (Table 1). Transcriptome completeness was assessed according to the BUSCO v.4.0.5 completeness score [29], and the test with eukaryota_odb10 showed 95.7% completeness in *C. pungelerii* and 93.7% completeness in *Bambalina* sp. (Table 1). Using these assemblies, we obtained 465 orthologous genes common to the *E. variegata* genome and produced a phylogenetic tree (Fig. 1a). The constructed bagworm phylogenetic tree showed early divergence of *C. pungelerii*, similar to previous studies [17]. Approximately 25,000 of the assembled contigs were assigned as protein-coding genes, and the BLAST search demonstrated that approximately 35% of the contigs were conserved between the two bagworms (Fig. 2a).

**Table 1:** Summary statistics of two bagworm transcriptome

Family	Psychidae	Psychidae
**Species**	*Canephora pungelerii*	*Bambalina* sp.
**Japanese name**	Kitakuro minoga	Kurotsuya minoga
**Sample information**		
Location	Yamagata Pref., Japan	Yamagata Pref., Japan
**Assembly statistics**		
# of contigs	99,286	68,365
Total length (bp)	79,974,576	64,049,463
Average scaffold length (bp)	805	936
Longest scaffold length (bp)	48,468	52,848
N50 (bp) (# of scaffolds in N50)	1815 (#11071)	2017 (#9196)
GC-content (%)	37.88	39.17
**BUSCO**		
Eukaryote	C:95.7%[S:76.5%,D:19.2%],F:1.6%,M:2.7%,n:255	C:93.7%[S:74.9%,D:18.8%],F:2.4%,M:3.9%,n:255

### Fibroin genes

**Fig. 3:**
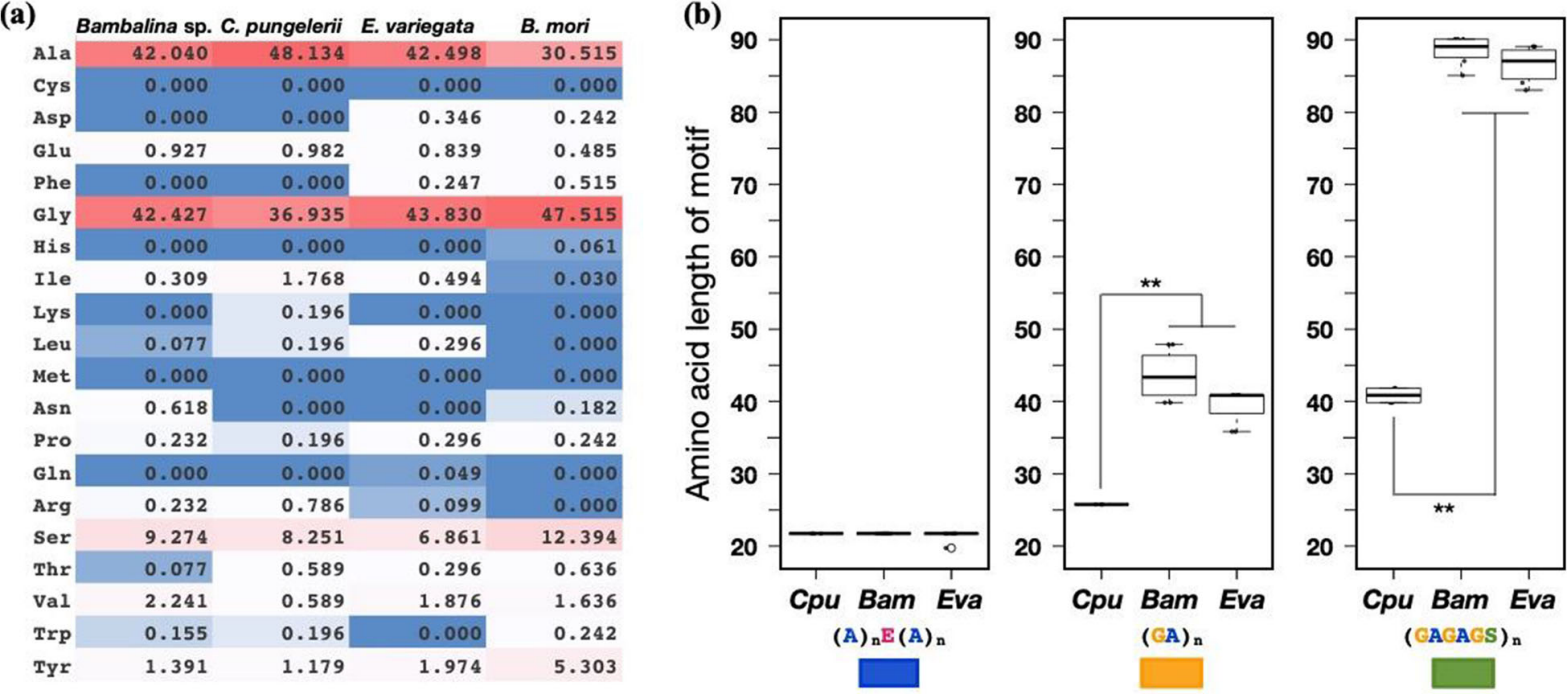
**a** Amino acid frequency of H-fibroin repetitive domains. The frequencies of *C. pungelerii* (*Cpu*) and *Bambalina* sp. (*Bam*) were significantly correlated (Pearson’s correlation coefficient = 0.98, *p* < 0.01). **b** Boxplot comparing the repeat lengths of each motif. (A) _n_E(A)_n_ showed a common length of 23 residues, while the GA motifs in *C. pungelerii* (*Cpu*) showed only half the length of those in other bagworms, regardless of whether they contained serine or not

Fibroin gene sequences were searched in the assembled transcriptome references based on *E. variegata* L-fibroin and H-fibroin sequences (GBP72856.1 and GBP83861.1), and the N/C-terminal regions and several repetitive units were obtained in *C. pungelerii* and *Bambalina* sp., respectively. The mRNA expression of the obtained L/H-fibroin genes was confirmed by cDNA-seq in each larval body, and both genes were highly expressed (Fig. 2b). Curated bagworm fibroins were well conserved in the three bagworms (Fig. 2c, d). The H-fibroin sequences of both N/C-terminal regions were very similar, and the features of a short C-terminal region were observed to be shared (Fig. 2d). The repetitive domains were also very similar among the three bagworms, with no significant differences in motif sequences or the distribution of the amino acid frequency (Fig. 3a). The unique combination of (A)_n_ and (GA)_n_ motifs observed in the repetitive domains of *E. variegata* was absent in other lepidopteran insects but was clearly apparent in *C. pungelerii* and *Bambalina* sp. H-fibroins. The separation of poly-A sequences by glutamic acid residues was also observed in the Psychidae family. Contrary to the sequence similarity of the terminal and repetitive domains, differences in the length of certain repetitive motifs were observed. The repetitive domain of bagworm H-fibroin is divided into four regions: an (A)_n_ motif region with a central glutamic acid (E), a pure (GA)_n_ motif region, a linker composed of isoleucine (I) and valine (V), and a (GA)_n_ motif region containing serine (S). Within these regions, the length of the two (GA)_n_ motif regions flanking the linker varied greatly among bagworms. The two (GA)_n_ motif regions in *E. variegata* and *Bambalina* sp. averaged 40 and 90 residues in length, respectively, whereas that of *C. pungelerii* was approximately half the size (Fig. 3b).

### Mechanical properties of bagworm silk

**Fig. 4:**
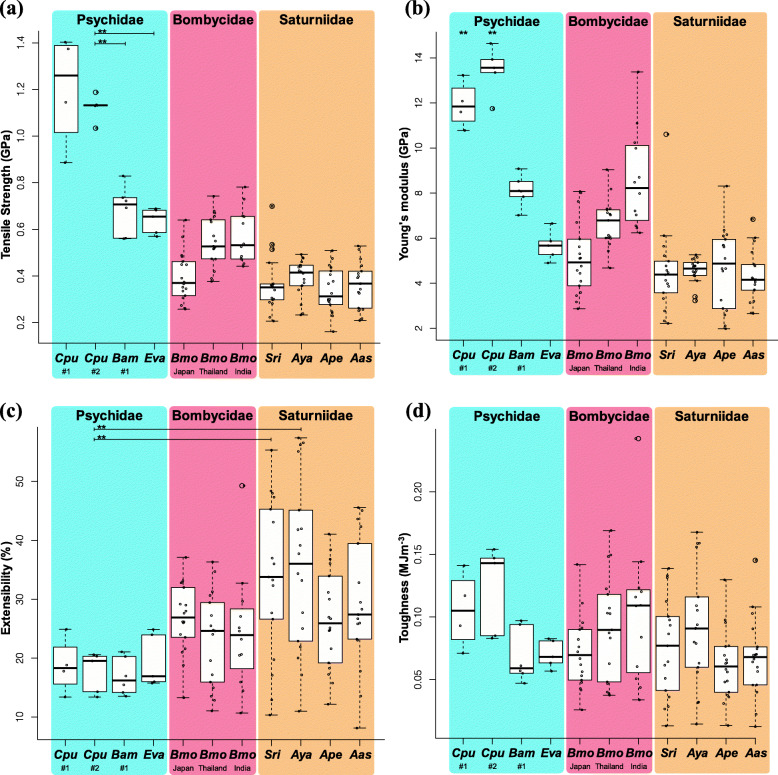
Mechanical properties, including tensile strength (**a**), Young’s modulus (**b**), extensitivity (**c**), and toughness (**d**), in each lepidopteran family, including Psychidae (*C. pungelerii* (Cpu), *Bambalina* sp. (Bam), and *E. variegata* (Eva)), Bombycidae (*B. mori* (Bmo), and Saturniidae: *S. ricini* (Sri), *A. yamamai* (Aya), *A. pernyi* (Ape), and *A. assama* (Aas)). The tensile strength and Young’s modulus of *C. pungelerii* silk were significantly higher than those of other lepidopteran silks (**: *p* < 0.01; t-test). Each plot represents a separate silk sample. The data for *Eva* are from [10], and *Bmo*, *Sri*, *Aya*, *Ape*, and *Aas* are from previous study [14]

The mechanical properties of the bagworm silks produced from fibroins with different motif lengths of (GA)_n_ motifs were examined by tensile tests. Using reeled silks from each bagworm bag, the following parameters were measured as mechanical properties: tensile strength (MPa), Young’s modulus (GPa), extensibility (%), and toughness (MJ/m^3^). These properties are summarized in Fig. 4. The newly calculated values for *C. pungelerii* and *Bambalina* sp. silks as well as that of *E. variegata*, showed higher tensile strength than has been found in other lepidopteran silks (Fig. 4a). Comparative analysis of the mechanical properties suggested that the length of the (GA)_n_ motifs may have an impact on the tensile strength and Young’s modulus (Fig. 4a, b). The strength of *C. pungelerii* silk, which is composed of short (GA)_n_ motif H-fibroin (Fig. 3b), was nearly twice as high as those other bagworm silks. The obtained tensile strength of 1.4 GPa and Young’s modulus of 1.3 GPa are currently the highest recorded among lepidopteran species.

**Fig. 5:**
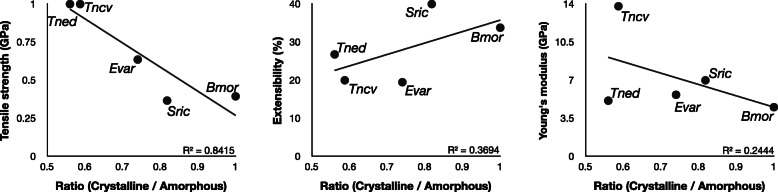
The summarized relationship between the balance of crystalline/amorphous regions and mechanical properties based on previous studies. The higher the ratio of crystalline regions is, the lower the tensile strength. The fibroin structure data of *Tncv* (*Trichonephila clavipes*), *Tned* (*Trichonephila edulis*), *Sric* (*Samia cynthia ricini*), and *Bmor* (*Bombyx mori*) were obtained from [28], and those for *Evar* (*Eumeta variegata*) were obtained from [12]. Each mechanical property value was taken from previous studies, *Tncv* [9]:, *Tned* [30]:, *Sric* and *Bmor* [14]:, *Evar* [10]:, respectively

On the other hand, motif length did not change other mechanical properties, such as extensibility or toughness. The potential impact of the motif length difference in H-fibroins on the mechanical properties was verified using secondary structure data. The protein secondary structures were obtained from synchrotron radiation measurement data [12, 28], and mechanical property data were taken from [9, 10, 14, 30]. Although the crystalline and amorphous region data for *C. pungelerii* and *Bambalina* sp. was not measured in this study, the analysis of the relationship between the ratio of crystalline and amorphous regions and the mechanical properties based on previous studies showed that only tensile strength decreased with the ratio of the crystalline region (Fig. 5). Hence, these results are meant only as a reference, but the balance of the crystalline/amorphous region has an impact on the tensile strength, which may explain the strength of the silk produced from *C. pungelerii* H-fibroin.

## Discussion

We prepared transcriptome references for *C. pungelerii* and *Bambalina* sp., which are closely related to genome-sequenced species *E. variegata,* and confirmed the conservation of the unique motifs in H-fibroins and the mechanical properties of bagworm silks among these species. The results strongly suggested that the bagworm-specific motif is the key to the characteristic mechanical properties of the bagworm silks.

The bagworm H-fibroin structure based on the motifs in the repetitive domain has been discussed previously. The WAXD and small-angle X-ray scattering (SAXS) patterns of *E. variegata* silk indicate that the poly-A and GA motifs correspond to crystalline and amorphous regions, respectively [12]. This structural model follows the stress-induced helix-to-sheet structural phase transition [31, 32]. In the fibres spun by bagworms, poly-A and pure (GA)_n_ motifs are responsible for forming β-sheets, and linker and (GA)_n_ motifs including serine are form the amorphous region [12]. As these repetitive units are assembled to form the hierarchical nanofibril structure, the motif balance is strongly related to the mechanical properties of bagworm silk. Therefore, the change in the balance of crystalline/amorphous regions may partially explain why *C. pungelerii* silk showed more than twice the tensile strength of other silks while maintaining the poly-A and linker sequence sizes. Incidentally, we should be cautious here about whether we can directly compare the data from previous studies. In particular, the mechanical properties are susceptible to the measurement methods, equipment, humidity, temperature, and even differences between individuals [33]. Figure 4 contains data from the previous studies [10, 14], all measured under the same conditions (EZ-LX universal tester with a 1 N load cell, at a strain rate of 10 mm/min at 25 °C and 48% relative humidity, see Methods). Any new comparisons in the future will need to be observed according to this standard.

Then, the question arises of why Oiketicinae silk has become stronger than that of other bagworms? The reason may have been to stably protect the larvae and eggs during the evolution of a wingless phenotype. All females of the subfamily Oiketicinae show a complete loss of wings in the pupal and adult stages. Wingless bagworm females are widely studied, and in many species, the wing discs that develop in the final larval stage degenerate during the prepupal or pupal stages through apoptosis [34, 35]. However, females of the subfamily Oiketicinae have lost the ability to develop wing discs during larval-pupal metamorphosis [36, 37]. Therefore, the subfamily Oiketicinae shows the most advanced established form of wing loss in the family Psychidae, and the independent clade is systematically supported [17]. Furthermore, within the Oiketicinae clade, *C. pungelerii* is phylogenetically divergent from *Bambalina* sp. and *E. variegata* (Fig. 1a). It is not well understood how different the lifestyle of *C. pungelerii* is from those of other species, but differences have been observed in terms of the shape and construction of the bag. Small flattened plant materials are often used to produce Bambalina bags (Fig. 1a). The bag is densely coated with such materials without gaps and therefore generally exhibits a hard consistency. On the other hand, in the bags of *C. pungelerii*, branches of varying lengths are used as surface materials. Because it is largely only the bases of these branches and leaves that are attached by silk threads (Fig. 1b, c), the bag surface is less dense and is generally soft and elastic [38]. In addition, the durability and stability required for *C. pungelerii* bags may be different from those of other bagworms. The development time of *E. variegata* is approximately 1 year, while that of *Canephora unicolor* (a close relative of *C. pungelerii*) is reported to be up to 730 days [7]. On the other hand, there are relatively few host plant families upon which *C. unicolor* hangs its bags. There are few potential host plants that can be selected in nature, and it is believed that the strength of the link with the host plant is the key to *C. unicolor* survival. Differences in bag material assembly and development time may have been the cause of the strong threads of *C. pungelerii*, even though the bag size is approximately four times smaller than that of *E. variegata*.

Omics analyses, such as genomic, transcriptomic, or proteomic analyses, can reveal the molecular background of protein-based materials. The search for new protein-based materials is currently becoming easier and faster, especially with the multiple standardization projects for genome information [39–43]. Comprehensive research allows comparative analysis by increasing the amount of curated information. The present study suggested that the percentage of crystalline regions may affect the mechanical properties of biomaterials. Such linkage of biochemistry and biophysics will contribute to the further development of the artificial design of highly functional structural proteins.

## Conclusions

Despite being closely related species, the silk threads of *C. pungelerii* and *Bambalina* sp. were a large difference in the mechanical properties. In particular, the tensile strength of *C. pungelerii* silk threads was much stronger than those of any other lepidopteran. This research has shown that one reason for this may be the balance of crystalline/amorphous regions in the H-fibroin repetitive domain, as shown by omics analysis. De novo transcriptome analysis has established their gene references, including new bagworm H-fibroin sequences, and the amorphous region of *C. pungelerii* was relatively short, which may have led to the high tensile strength. Our suggestion about the relationship between structural balance and the mechanical property of a structural protein will be helpful for future biomaterial research and industrial applications.

## Data Availability

The raw sequence reads used for de novo assembly and expression analysis were submitted to DDBJ SRA (sequence read archive). The accession numbers of the raw sequence reads are DRR227299 (*C. pungelerii*) and DRR227298 (*Bambalina* sp.). The assemblies are available in DDBJ under accession numbers ICRH01000001-ICRH01099286 (*C. pungelerii*) and ICRG01000001-ICRG01068362 (*Bambalina* sp.). Each L/H-fibroin gene sequence is submitted as ICRH01099286 (*C. pungelerii* L-fibroin), ICRG01068362 (*Bambalina* sp. L-fibroin), ICRH01099284-ICRH01099285 (*C. pungelerii* H-fibroin), and ICRG01068360- ICRG01068361 (*Bambalina* sp. H-fibroin).
